# Factors influencing the choice of urethral slings over artificial sphincter for male stress urinary incontinence

**DOI:** 10.1007/s11255-025-04444-y

**Published:** 2025-03-14

**Authors:** Zachary Boston, Kunj Jain, Hassan Choudhry, Meher Pandher, Aleksandar Popovic, Amjad Alwaal

**Affiliations:** 1 Division of Urology, Department of Surgery, Rutgers New Jersey Medical School, Ambulatory Care Building, 140 Bergen Street, G-Level, Room 1680, Newark, New Jersey 07103 USA; 2 Rutgers New Jersey Medical School, 185 South Orange Avenue, Newark, New Jersey 07103 USA

**Keywords:** Artificial urethral sphincter, Urethral slings, Stress urinary incontinence

## Abstract

**Objective:**

To examine the factors influencing urologists’ decision to offer slings instead of AUS for managing male stress urinary incontinence.

**Methods:**

The American College of Surgeons National Surgical Quality Improvement Program (NSQIP) database 2006–2021 was used to identify patients undergoing surgical procedures for male urinary incontinence using current procedural terminology (CPT) codes. The Current procedural terminology (CPT) codes for AUS (53,445) and male slings (53,440) were used to analyze the data appropriately. The cases with incomplete demographic data were excluded. Patient characteristics of interest were race, age, smoking status, obesity, HTN, COPD, ASA classification, use of glucocorticoids, history of cancer, and diabetes mellitus. Chi square and multivariate logistic regressions were used to identify significant predictors of outcomes. Significance was defined as p<0.05.

**Results:**

Among 4098 patients, 2407 underwent AUS implantation, and 1691 received a sling for male SUI. African American males were significantly more likely than Caucasian males to receive a sling (OR = 5.566, *p* < 0.05). The patients with comorbidities such as diabetes mellitus, hypertension, use of glucocorticoids, cancer, increased ASA, and history of DVTs are significantly more likely to undergo sling placement. COPD, congestive heart failure, and dialysis had no impact on the choice of male urinary incontinence management.

**Conclusion:**

Male patients who are African American or have comorbidities such as history of diabetes, hypertension, cancer, DVT, and glucocorticoid use were more likely to be offered slings for stress urinary incontinence. These findings suggest a preference among urologists to recommend slings for patients with higher surgical risk profiles.

## Introduction

Urinary incontinence can be defined as the involuntary leakage of urine and can significantly impact quality of life and daily function. [1] Stress urinary incontinence (SUI), which is urine leakage that occurs due to activities that increase abdominal pressure, can present during coughing, sneezing, and exercising. [2, 3] Initial treatment for mild symptoms involves conservative options such as utilizing incontinence pads. [4] Although effective in managing symptoms, these pads can be inconvenient and socially stigmatizing for many men. [5] Alternatively, artificial urinary sphincter (AUS) implants and urethral sling placement are surgical treatment options that can correct urinary incontinence.

AUS is considered the gold standard for the treatment of male SUI. However, male sling over recent decades has become increasingly popular. With the less invasive nature of slings, patients and urologists have shown an increased preference for slings, likely due to the well-known complications that can arise from AUS implants. [6] The development of various models of slings can provide incontinence relief using different mechanisms of action. This allows modern slings to be effective in treating the varying types of pathophysiology that drive incontinence, as each model can address different degrees of detrusor contractility and urine leakage. [6]

There remain several factors that urologists may need to consider when deciding which surgical technique to pursue when treating SUI, including preoperative patient characteristics and postoperative success. [7] For example, urethral slings have been shown to have better postoperative success for mild to moderate cases of SUI. [8] There is a growing body of literature detailing the degree of urine leakage, proximal urethral mobility, prior radiation therapy or incontinence surgery, and detrusor contractility as the driving factors in deciding which procedure is optimal. [6] However, the influence that other preoperative patient characteristics, such as chronic health conditions and social determinants of health, have in preference for a sling over an AUS implant is still largely debated. Factors such as metabolic disease, cardiovascular disease, and socioeconomic demographics each have the capacity to worsen SUI. These chronic health comorbidities, such as metabolic disease, cardiovascular disease, cancer and neurogenic conditions, are known to be associated with impaired bladder function. [9] Previous studies have highlighted the advantages of urethral slings, including being independent of mechanical components, less invasive, associated with lower complication rates, more cost-effective, and offering quicker recovery times compared to AUS. [10–12] Additionally, the lower initial acquisitional cost of a sling compared to AUS devices highlights the potential impact that socioeconomic status may have on patients when selecting surgical options.

There is a paucity of research that elaborates on the impact that other patient-level factors, like chronic health status and social vulnerability, may have on preference for a male sling over AUS. This study aims to analyze these potential factors further and identify additional considerations that guide urologists in offering slings over AUS for male SUI. Understanding these factors can enhance treatment decision-making, optimize patient outcomes, and improve healthcare delivery.

## Materials and methods

A retrospective study was conducted to evaluate male urinary incontinence cases from 2006 to 2021. The American College of Surgeons National Surgical Quality Improvement Program (NSQIP) was utilized to identify these patients who were surgically operated on for AUS or urethral sling procedures. The Current procedural terminology (CPT) codes for AUS (53,445) and male slings (53,440) were used to analyze the data appropriately.

The patient demographic variables from NSQIP were examined including age, smoking status, obesity, hypertension (HTN), chronic obstructive pulmonary disorder (COPD), and American Society of Anesthesiologists (ASA) classification. To ensure consistency in gathering the data, only cases with complete demographic data made available were incorporated in the data. Further recording of medical history includes patient use of glucocorticoids, history of cancer, and diabetes mellitus status. Patients with missing data were excluded from the study. No other exclusion criteria was used.

Statistical analyses were applied to analyze patient outcome data accordingly including Chi-square analyses and multivariable logistic regression models. Odds ratios and confidence intervals were also calculated. For all conducted statistical tests, *p* values less than 0.05 indicated significance in the results. All data were recorded in Microsoft Excel and analyzed in SPSS Statistics.

## Results

NSQIP revealed 4098 cases of urinary incontinence (excluding cases with any missing patient demographic data) in males that was surgically treated with AUS or slings. Of these cases, 2407 patients received an AUS and 1691 patients received a urethral sling. AUS surgery data served as the reference values in comparison to sling surgeries when calculating odd ratios, confidence intervals, and *p* values. African American men were 5.566 times (CI: 1.528–20.274) more likely to be treated with slings than AUS (Table 1). Confidence intervals showed that urologists were up to 20 times more likely to offer sling surgeries to patients compared to AUS implants (Table 1).

**Table 1:** Predictors of male urethral sling surgeries

	Race (African American)	Insulin dependent diabetes	HTN^a^	Glucocorticoid use	Cancer
	OR^b^ (95% CI^c^)	OR^b^ (95% CI^c^)	OR^b^ (95% CI^c^)	OR^b^ (95% CI^c^)	OR^b^ (95% CI^c^)

Male urinary incontinence surgery					
AUS	Ref^d^	Ref^d^	Ref^d^	Ref^d^	Ref^d^
Sling	5.566 (1.528–20.274)*	3.521 (1.458–7.752)*	1.471 (1.294–1.674)*	2.295 (1.445–3.645)*	3.646 (1.705–7.796)*

^a^Hypertension

^b^Odds Ratio

^c^Confidence interval

^d^Reference

^*^Significance, defined as p < 0.05

Furthermore, male patients with a history of insulin-dependent diabetes, HTN, glucocorticoid use, and cancer were all more likely to have sling surgeries by 3.521 (CI: 1.458–7.752), 1.471 (CI: 1.294–1.674), 2.295 (CI: 1.445–3.645), and 3.646 (CI: 1.705–7.796) times respectively (Table 1). All predictor variables had calculated odd-ratio values that were statistically significant (*p* value < 0.05) and greater than one when utilizing AUS surgeries as reference values (Table 1). Moreover, patients with increased ASA and a history of deep vein thrombosis (DVT) were also shown to be more likely to have sling surgeries. Diagnoses such as COPD, congestive heart failure, and dialysis had no impact on male stress urinary incontinence management.

## Discussion

Our findings indicate that male patients who are African American or have increased comorbidities were more likely to be offered slings over AUS for treating stress urinary incontinence. This aligns with a prior study conducted by *Godwin et al.,* who demonstrates that age (older > 80), diabetes, history of transient ischemic attack, and coronary artery disease are significant patient factors that increase rates of infection and erosion after undergoing AUS placement. [22] Using a univariate analysis, the hazard ratios in this study for patient age, history of TIA, diabetes mellitus, and CAD were 1.06 (CI: 1.02 - 1.11; *p* = .005), 2.49, (CI: 1.14 - 5.44; *p* = .02), 1.84 (CI: 1.07 - 3.19; *p* = .03), and 2.48 (CI: 1.01 - 1.53; *p* = .002), respectively. Additionally, Garetto et al*.* highlights that AUS implantation was associated with higher-grade complications, reinterventions, and device explantations compared to a modern sling (*p* = 0.03).[21] Our data support both of these previous studies, as patients with comorbidities, such as a history of diabetes, hypertension, cancer, DVT, and glucocorticoid use are likely to have fewer complications with sling as the treatment method for stress urinary incontinence.

Within this study, male patients with comorbidities such as insulin-dependent diabetes, corticosteroid use, and hypertension were shown to be more likely to be offered slings than AUS implantation for addressing urinary incontinence. Despite being historically considered the gold standard for SUI treatment, AUS surgery is accompanied by high-grade complications, such as urethral erosion, urethral atrophy and mechanical failure. [25] Sling procedures are less invasive than AUS and have minimal or self-limiting complications in comparison. [25] This data support a study conducted by Viers et al*.*, which found that the presence of diabetes was associated with a 2.3 fold increase in AUS erosion and infection (*p* = 0.025). [24] It is well known that patients with diabetes are at an increased risk for both preoperative and postoperative complications. Decreased wound healing capacity, increased blood viscosity, and inflammatory processes can put diabetic patients at an increased risk of postoperative complications and hospitalization after undergoing AUS. [15, 23] Similarly, corticosteroid use can also impair wound healing and tissue integrity.[16] According to Clark et al., the patients with exposure to steroid therapy have a higher rate of AUS reoperation (p = 0.04). Hypertension compromises the integrity of the vascular system, which could pose issues for complex surgical procedures that are reliant on sustaining tissue integrity, such as AUS devices.[17] Kretschmer et al. analyzed several different potential risk factors for AUS failure. An important finding in this study was that patients who required perioperative anticoagulants were at a significantly increased risk of AUS device failure. [26] Patients who needed any anti-platelet therapy or vitamin K antagonist, likely needed treatment for compromised cardiovascular health. While the details of each patient’s cardiovascular health status were not disclosed, any impairment to microvascular blood supply is detrimental to urethral healing. [26] Additionally, the stress that hypertension, deep vein thrombosis, and other chronic arterial diseases put of the vascular system can ultimately lead to damage to the AUS device. [18] Uncontrolled diabetes, hypertension or steroid exposure in the setting of poor tissue viability and healing capacity can result in several AUS postoperative complications such as device erosion and infection. Patients with these comorbidities should be considered high risk for AUS device implantation surgery because there is a higher likelihood of complications. The explanation for our findings is likely because urologists are more willing to offer slings as a result of their decreased risk of complications, especially in patients with chronic comorbidities. Despite substantial evidence supporting the success rates of both AUS and sling techniques, understanding the effects that comorbidities have on patients may better optimize overall SUI treatment.

In this study, we found patients who identified as African American as a predictive factor for the offered surgical treatment of SUI. Although some prior studies have suggested that race may serve as a predictor for outcomes following urologic procedures, there has been extensive conflicting research that indicates race alone does not affect the postoperative complication rate. Indeed, it is the multi-factorial interplay of racial disparities in healthcare and patient comorbidities that have a greater impact on surgical outcomes. [13] While our study provided statistical significance indicating an association between African Americans and the technique used to treat SUI, the explanation for this is likely due to a culmination of socioeconomic factors and healthcare disparities, to which race is also linked.

While there is no conclusive research that establishes a direct reason for a sling should be preferred over an AUS for surgical treatment of SUI in African American men, our results may be due to the nature of each surgical procedure. AUS is a more invasive surgery that has well-known associated postoperative risks, including urethral injury and persistent SUI. [27] As previously stated, the patients with chronic comorbidities that impact microvascular blood supply to the surrounding urethral surgical site, such as cardiovascular disease and diabetes, are at a significantly increased risk for AUS device failure. [26] Furthermore, it is well understood that patients with lower socioeconomic status and healthcare access, specifically African Americans, have a higher prevalence of cardiovascular and metabolic comorbidities.[14] These medical risks alone, may be significant enough for urologists to offer male slings over AUS to African American men. Because AUS procedures have a higher complication rate, it is feasible to consider the explanation for our findings as a result of the impact that confounding sociodemographic factors have on developing comorbidities for African American patients.

Additionally, the initial cost-effectiveness of receiving a sling over AUS highlights the potential role that socioeconomic status and social determinants of health may have had on the results of our study. AUS implants often have a higher upfront cost, as the longer operative time and prevalence of post-operative complications can lead to an increased financial burden. [25] This may deter patients from marginalized populations from electing to receive an AUS implant. However, this study did not have the severity of each patient’s urinary incontinence available for analysis. It is possible that African American men did not have the severity of SUI compared to other races. In other words, they did not meet the SUI severity requirements to be offered AUS. However, there is a possibility that many African American men in our study were offered AUS less often due to an established physician bias in treating SUI. Physicians may be less inclined to offer AUS to African American patients due to the additional associated risk, as well as the short-term financial burden According to Anger et al., black patients are less likely to receive coronary bypass, knee arthroscopy, or hysterectomy compared to other races, even when indicated. [28] This aligns with a common pattern seen throughout medicine, as socioeconomically disadvantaged individuals are faced with limitations in access to treatment and are subjected to bias throughout the healthcare system. Our study highlights the need for additional patient-level and system-level analysis of the social determinants that impact the relationship between race and surgical treatment of SUI.

Furthermore, our study suggests that patients who have a cancer diagnosis are more likely to be offered a urethral sling procedure over AUS. This aligns with a study conducted by Mamane et al., which indicates that radiotherapy prior to AUS device placement can lead to increased instances of AUS erosion, infection and explantation. [19] In this study, 26.5% of the 437 patients who previously had radiation therapy experienced AUS device erosion, compared to 14.8% of non-radiated individuals. Radiation can result in local tissue fibrosis and decrease vascularization to the treated region. Poor tissue integrity can compromise the normal tissue healing process and reduce local tissue response to infection, ultimately leading to AUS device mechanical failure. [19] However, Ghaffar et al. analyzed the correlation of mid-urethral slings and radiotherapy history. This study found significantly greater odds of infection (odds ratio = 3.06, 95%, *P* < .001) and explanation (odds ratio = 2.93, *P* < .001) in patients who received mid-urethral sling procedures prior to radiation therapy. [20] Despite these findings, the study done by Ghaffar et al. was limited by sample size. Therefore, the explanation for lower sling success rates for patients receiving radiotherapy remains inconclusive. While there was limited data regarding the cancer etiology and details of exposure to radiation therapy for our sampled cohort, the Mamane et al*.* study provides a possible explanation of our data findings. Further work in analyzing the radiation history of patients prior to AUS and sling implantation could provide insight to urologists electing to use surgical treatment in cancer patients who have SUI.

While clear results were obtained from the analyses performed, it is essential to acknowledge some limitations of the study. NSQIP has a limited scope of data to some extent since there are postoperative outcomes included only up to 30 days. Therefore, follow-up data on patients such as if they needed multiple surgeries or switched from a sling surgery to AUS afterward was not known due to the retrospective nature of the database. There is also no data from NSQIP regarding the severity grading of stress urinary incontinence. Because of this, we were unable to have complete standardization when comparing the data. Additionally, only hospitals enrolled in NSQIP will be part of the dataset and most of these hospitals tend to be larger and wealthier institutions which could result in an unrepresentative sample of underserved areas. Lastly, NSQIP data are not randomized but is retrospective and observational, which does not account for the possibility of confounding variables affecting the data.

The intersection between comorbidities and socioeconomic demographics is seen in various facets of the healthcare system. While these factors that were found to be significant within our study seem distinct from one another, indeed, they likely have a causal relationship. To address the financial burden and bias that marginalized populations face, a system-level introspective analysis is necessary. Much research focuses on the patient-level factors that contribute to health outcomes, however, system and facility-level characteristics need to be evaluated to have a complete understanding of the disparities within our healthcare system. An area of intervention in determining SUI surgical treatment can begin with establishing a standardization within the process. Urologists may benefit from having explicit guidelines or standards in the surgical decision-making of SUI treatment that encompass a thorough history and physical exams, socioeconomic factors, and chronic health status.

## Conclusion

Male patients who are African American or have increased comorbidities such as history of diabetes, hypertension, cancer, DVT, and glucocorticoid use were more likely to be offered slings for stress urinary incontinence. These findings highlight a tendency among urologists to recommend slings for patients with higher surgical risks, emphasizing the need for personalized treatment strategies that consider both medical and sociodemographic factors. 

## Data Availability

No datasets were generated or analysed during the current study.
